# Integrating single-cell RNA-seq, bulk RNA-seq and network pharmacology reveals protective effect of salidroside in peritoneal dialysis-associated peritoneal fibrosis

**DOI:** 10.3389/fphar.2025.1558366

**Published:** 2025-06-20

**Authors:** Shuting Li, Yue Ji, Silin Zhu, Mi Liu, Dan Luo, Qimei Luo, Min Mo, Haibo Long, Fenfen Peng, Zhanjun Jia, Xianrui Dou

**Affiliations:** ^1^ Department of Nephrology, The Eighth Affiliated Hospital of Southern Medical University (The First People's Hospital of Shunde Foshan), Foshan, China; ^2^ Department of Nephrology, Zhujiang Hospital, Southern Medical University, Guangzhou, China; ^3^ Nanjing Key Laboratory of Pediatrics, Children’s Hospital of Nanjing Medical University, Nanjing, China

**Keywords:** salidroside, peritoneal dialysis, peritoneal fibrosis, single-cell RNA, network pharmacology

## Abstract

Salidroside (2- (4-Hydroxyphenyl) ethyl β-D-glucopyranoside, SAL) is a bioactive compound present in Rhodiola rosea L., exhibiting diverse pharmacological properties such as anti-inflammatory and anti-fibrotic effects. Despite its known benefits, the therapeutic potential of SAL in peritoneal dialysis (PD) -induced peritoneal fibrosis remains unexplored. This study aims to investigate the protective effects of SAL in PD-related peritoneal fibrosis and its underlying mechanisms through the integration of single-cell RNA-seq, bulk RNA-seq, and network pharmacology analyses. A total of 249 disease targets were identified through single-cell RNA-seq and bulk RNA-seq analyses. Functional enrichment analysis highlighted the involvement of extracellular matrix organization, neutrophil degranulation, and the vitamin D receptor (VDR) pathway in peritoneal fibrosis. By intersecting 148 drug targets with the 249 disease targets, four therapeutic targets for SAL treatment against peritoneal fibrosis were pinpointed: cathepsin S, VDR, plasminogen activator urokinase, and galectin 3. In a murine model of peritoneal fibrosis induced by intraperitoneal injection of 4.25% PD fluid, SAL treatment significantly mitigated peritoneal fibrosis, as evidenced by reduced collagen deposition, decreased protein expression of α-smooth muscle actin and Collagen I, and a thinner peritoneum. *In vitro* experiments demonstrated that SAL treatment inhibited extracellular matrix deposition, potentially through upregulation of VDR expression. In conclusion, SAL may target VDR domains as a therapeutic agent for PD-related peritoneal fibrosis. These findings comprehensively identify potential therapeutic targets for SAL in combating peritoneal fibrosis, providing a theoretical basis for the clinical application of SAL in the treatment of peritoneal fibrosis.

## Background

Peritoneal dialysis (PD) is utilized worldwide as a modality for kidney replacement therapy for patients with end-stage renal disease (Himmelfarb et al., 2020). The efficacy of PD relies on the intact structure and function of the peritoneal membrane (Pletinck et al., 2012). However, chronic exposure to biological incompatibility of PD solution is one of the principal causes of ultrafiltration failure (UF). It has been demonstrated that prolonged PD exceeding 5 years can lead to morphological alterations, notably the loss of mesothelium, vasculopathy, and interstitial fibrosis (Krediet and Parikova, 2022). Consequently, the development of progressive peritoneal fibrosis is a prevalent complication in long-term PD patients. Peritoneal fibrosis is associated with compromised solute clearance, UF, and alterations in peritoneal membrane transport characteristics, thereby impacting the membrane’s functional capacity (Krediet and Parikova, 2022). There are few effective measures that could prevent and treat progressive peritoneal fibrosis. Deciphering the underlying pathogenesis and identifying novel treatment options are essential for PD patients.

The advancement of single-cell RNA sequencing technology and associated data analysis techniques has presented a unique opportunity to elucidate the molecular features of PD-associated peritoneal fibrosis (Hu et al., 2023; Zhang J. et al., 2021). Previous research has indicated that data obtained from single-cell RNA sequencing supports the significant involvement of peritoneal mesothelial cells in the pathogenesis of peritoneal fibrosis (Huang et al., 2023). Peritoneal mesothelial cells act as the initial line of defense against high glucose PD fluid, contributing significantly to solute transport, immune surveillance, and maintenance of serosal homeostasis (Si et al., 2019). Moreover, these cells play a pivotal role in orchestrating the peritoneal response to injury and inflammation (Mo et al., 2023). This study utilizes single-cell RNA sequencing to generate individual cell transcriptome profiles from PD specimens, focusing on peritoneal mesothelial cells.

In recent years, network pharmacology has emerged as a systematic approach to drug discovery, transforming the comprehension and management of diseases (Nogales et al., 2022). It surpasses the constraints of organ-centric and single-target therapy approaches, allowing for precise and efficient therapeutic interventions. Numerous studies have employed network pharmacology methodologies to elucidate the mechanisms by which drugs act on diseases (Shang et al., 2023; Zhou et al., 2022; Chen Y. et al., 2022).

The perennial herbaceous plant genus *Rhodiola*, primarily found in cold, high-altitude regions of Asia and Eastern Europe, has a history of over a thousand years in traditional Tibetan medicine (Panossian et al., 2010). Recorded in the Tibetan monograph “Jing Zhu Materia Medica” (Dge-bśes D-d, 1745–1840 AD), *Rhodiola* has been utilized to address symptoms such as cough, hemoptysis, fever, pain, bruising, and other injury- and inflammation-related conditions (Tao et al., 2019). *Rhodiola* was officially included in the Chinese Pharmacopoeia for therapeutic use in 1977 (Chinese Pharmacopoeia Commission, 2015). Furthermore, it was acknowledged by the European Medicines Agency for human consumption in 2012. Salidroside (SAL, p-hydroxyphenethyl-β-D-glucoside; C14H20O7; 300.30), recognized as a potent bioactive compound found in *Rhodiola* species, exhibits diverse pharmacological activities in both *in vitro* and *in vivo* studies, including anti-hypoxic, antioxidant, anti-inflammatory, renal protective effects, and a high level of safety (Liu et al., 2023; Zhang X. et al., 2021; Liang et al., 2023). SAL has been shown to improve fibrosis in various organs, such as the heart (Chen H. et al., 2022; Hai et al., 2023), lung (Tang et al., 2016), liver (Hu et al., 2021) and kidney (Li R. et al., 2019; Xue et al., 2019), through diverse signaling pathways, including the inflammatory pathway. However, the potential therapeutic effectiveness of SAL in PD patients with end-stage renal disease remains uninvestigated.

This study aims to investigate the protective effects of SAL in PD-related peritoneal fibrosis and its underlying mechanisms through the integration of single-cell RNA-seq, bulk RNA-seq, and network pharmacology analyses. The accuracy of the predictions was verified through *in vivo* testing utilizing a mouse model of peritoneal fibrosis and *in vitro* experiments involving the treatment of the cultured human mesothelial cell line (Met5-A cells) with transforming growth factor-β1 (TGF-β1) or high glucose. The current study will offer novel insights into the potential of SAL as a new therapeutic agent for improving peritoneal fibrosis.

## Methods and materials

### Single-cell RNA-seq analysis

Single-cell RNA-seq was conducted using data from the Gene Expression Omnibus database (GSE248762) to analyze cell composition and transcriptome features in effluent samples from PD patients with short vintage without UF (SV-NOT-UF) and those with long vintage and UF (LV-UF). The study included six SV-NOT-UF patients and four LV-UF patients. After applying data integration filters, we utilized the “NormalizeData” function from the Seurat package to standardize the expression values. Subsequently, the “FindVariableFeatures” function was employed to pinpoint 2,000 highly variable genes, which were then normalized using the “ScaleData” function. Dimensionality reduction of the identified highly variable genes was performed using the “RunPCA” function in Seurat. Data integration was achieved by establishing 2,000 anchors through the “FindIntegrationAnchors (RunHarmony)” feature. Clustering of the dimensionally reduced data was carried out using the “FindNeighbors” and “FindClusters” functions in Seurat. For improved visualization of the clustering outcomes, we implemented the T-distributed random nearest neighbor embedding (t-SNE) method, which combines T-distribution and random nearest neighbors. The clustering visualization was efficiently achieved by utilizing the “RunTSNE” function in the Seurat package with parameters set to dims = 1:25 and resolution = 0.7. Cell annotation, differential expression genes (DEGs) identification, and marker gene identification were conducted using reference datasets. Differential gene analysis was performed by employing the “FindAllMarkers” function in the Seurat package with criteria set at |log2FoldChange|>1 and adjusted *P* value < 0.05. Subsequently, the identification of marker genes specific to each cell type was carried out using the “FindAllMarkers” function in the Neuat software package. GO functional enrichment analysis and KEGG pathway enrichment analysis were conducted using the clusterProfiler package (version 3.14.3) for annotations and analyses. Visualization of the results was accomplished using the ggplot2 software package.

### Bulk RNA-seq analysis

The RNA-seq dataset of the human peritoneum was sourced from the Gene Expression Omnibus database (GSE62928) using the GEO query package in R software, version 4.04 (Davis and Meltzer, 2007). The dataset comprised four encapsulating peritoneal sclerosis (EPS) samples obtained from peritoneal biopsies of EPS patients undergoing PD, and four control (CTL) samples from uremia patients who had abdominal surgery for non-peritoneal conditions (n = 2) or underwent the initial implantation of a PD catheter (n = 2). DEGs between EPS and CTL were obtained using the DESeq2 R package. Genes with an adjusted *P*-value <0.05 were assigned as differentially expressed. A volcano map was generated using ggplot2 plotting and heatmap library in R software. KEGG pathway analysis was performed and visualized using Metascape at https://metascape.org.

### Drug targets of SAL

The molecular structure of SAL was obtained from PubChem (https://pubchem.ncbi.nlm.nih.gov/). The SuperPred database, Similarity Ensemble Approach (SEA), and Swiss Target Prediction were employed in the determination of SAL target genes. In order to enhance prediction accuracy, the data from the aforementioned databases were combined and duplicate entries were subsequently eliminated.

### Molecular docking validation of the binding capacity between SAL and targets

The three-dimensional structure of SAL was obtained from PubChem and refined for energy optimization using Chem3D software. Crystallographic coordinates were acquired from the Protein Data Bank or predicted through AlphaFold2.3. Preceding docking, PyMol 2.5 was utilized to eliminate solvent, hydrogen atoms, and ligands from the crystal structure. Docking was conducted with the entire active site considered. Both ligand and receptor were converted to PDBQT format via ADFRsuite 1.0. AutoDock Vina 1.5.6 was utilized for the docking calculations. The conformation with the highest total score was selected, followed by analysis and visualization using PyMOL 2.5 and UCSF ChimeraX 1.7.1.

### Animal model

As previous reported (Li S. et al., 2023), a mouse peritoneal fibrosis model was developed through daily intraperitoneal administration of 3 ml of standard PD fluid containing 4.25% glucose (Baxter Healthcare, Deerfield, IL) for 4 weeks. Briefly, eight-week-old male C57BL/6J mice were procured from the Southern Medical University Animal Center in Guangzhou, China. To investigate the role of SAL in peritoneal fibrosis, three groups of mice were treated as follows: (1) the control group received daily intraperitoneal injection of 3 ml saline and daily intragastric administration of saline (80 mg/kg), n = 6; (2) mice received daily PD fluid and daily intragastric administration of saline (80 mg/kg), n = 6; (3) mice received daily PD fluid and daily intragastric administration of SAL (80 mg/kg), n = 6. After the 28-day PD fluid treatment, the mice were sacrificed. Parietal peritoneal tissues were obtained for Masson staining and immunohistochemical staining as previously described (Li S. et al., 2023). Visceral peritoneal tissues were obtained for subsequent western bolt analysis. Antibodies used were described in Supplementary Table S1. The experimental protocol involving these animals was scrutinized and sanctioned by the Animal Experimental Ethics Committee at Southern Medical University (Approval No. LAEC-2020-166).

### Cell culture

Human peritoneal mesothelial cell line Met5-A was obtained from the Key Laboratory of Nephrology at the Third Affiliated Hospital of Sun Yat-Sen University in Guangzhou, China. Culturing was conducted using M199 (Gibco) supplemented with 10% fetal bovine serum, 1% Insulin-Transferrin-Selenium (Gibco), and 1% antibiotics. The cells were then maintained at 37°C in a 5% CO2 environment. Upon reaching 70% confluence, they were serum-starved for 12 h and then were exposed to 5 ng/ml of recombinant human TGF-β1 (R&D Systems, Minneapolis, MN) or d-glucose (Life Technologies, United States,138 mmol/L) for 48 h. In certain experiments, cells were treated with 50 μM SAL (Sal, Y-N0109, MedChemexpress, New Jersey, United States) for 48 h.

### Western blot analysis

Protein samples were obtained from tissues or cells by employing RIPA lysis buffer (KeyGen, Nanjing, China) as per established procedures. Protein concentrations were quantified using the BCA kit (Thermo Fisher Scientific, Waltham, MA, United States). Details of the antibodies used in the western blot analysis can be found in Supplementary Table S1.

### Immunofluorescence staining

Cells cultured on coverslips were fixed with 4% formaldehyde and permeabilized using 0.1% Triton X-100. Following a 1-h blocking step with 5% BSA, the specimens were subjected to overnight incubation at 4°C with primary antibodies. This was succeeded by a 1-h exposure to fluorescent-labeled secondary antibodies and subsequent staining with 4′,6-diamidino-2-phenylindole (DAPI) (BestBio, Shanghai, China). Imaging was performed using a confocal microscope (Nikon, Tokyo, Japan), with details of the antibodies utilized provided in Supplementary Table S1.

### RNA extraction and quantitative real-time-PCR

Total RNA was extracted from cell samples or tissues using Trizol reagent and reversed transcribed into cDNA following the manufacturer’s protocol. The resulting cDNA was then utilized in quantitative real-time PCR using the BIO-RAD Sequence Detection System. Primers were custom-designed and synthesized by Sangon (Shanghai, China), with their sequences provided in Supplementary Tables S1, S2.

### Cell viability assay

Met5-A cells were incubated with different concentrations of SAL for 48 h. Then, 10 μL per well of cck8 was added for 4 h. The absorbance was measured at 490 nm.

### Statistical analyses

The data were presented as mean ± SEM and analyzed utilizing SPSS 22.0 (SPSS Inc., Chicago, IL). Independent-Student’s t-test was used to compare two groups. One-way ANOVA followed by Least significant difference (LSD) test or Dunnett’s T3 procedure for multiple comparison was used for groups of three or more. A significance level of *P* < 0.05 was utilized for statistical analysis.

## Results

### Identification of disease targets

**FIGURE 1:**
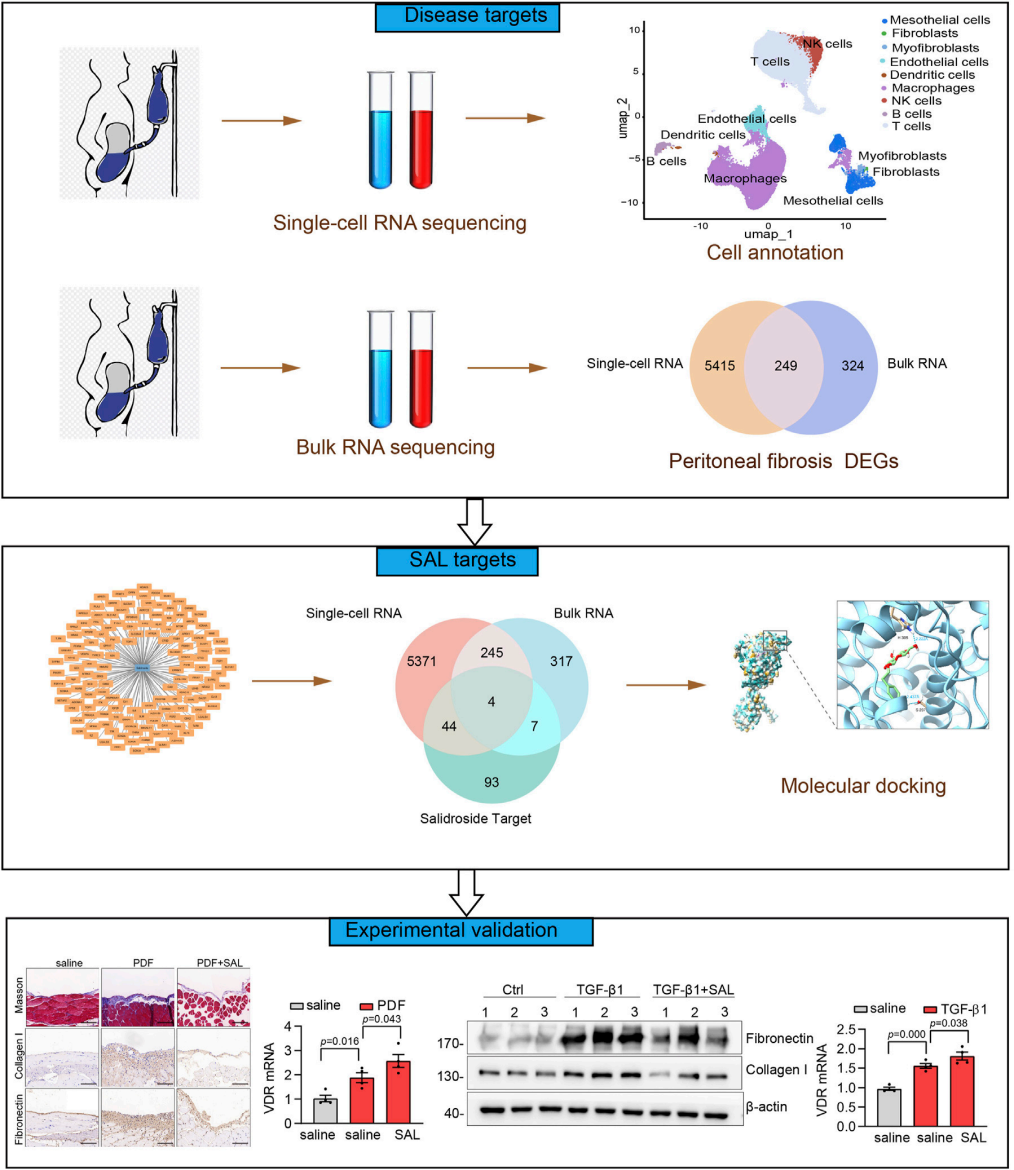
Flowchart of this study.

**FIGURE 2:**
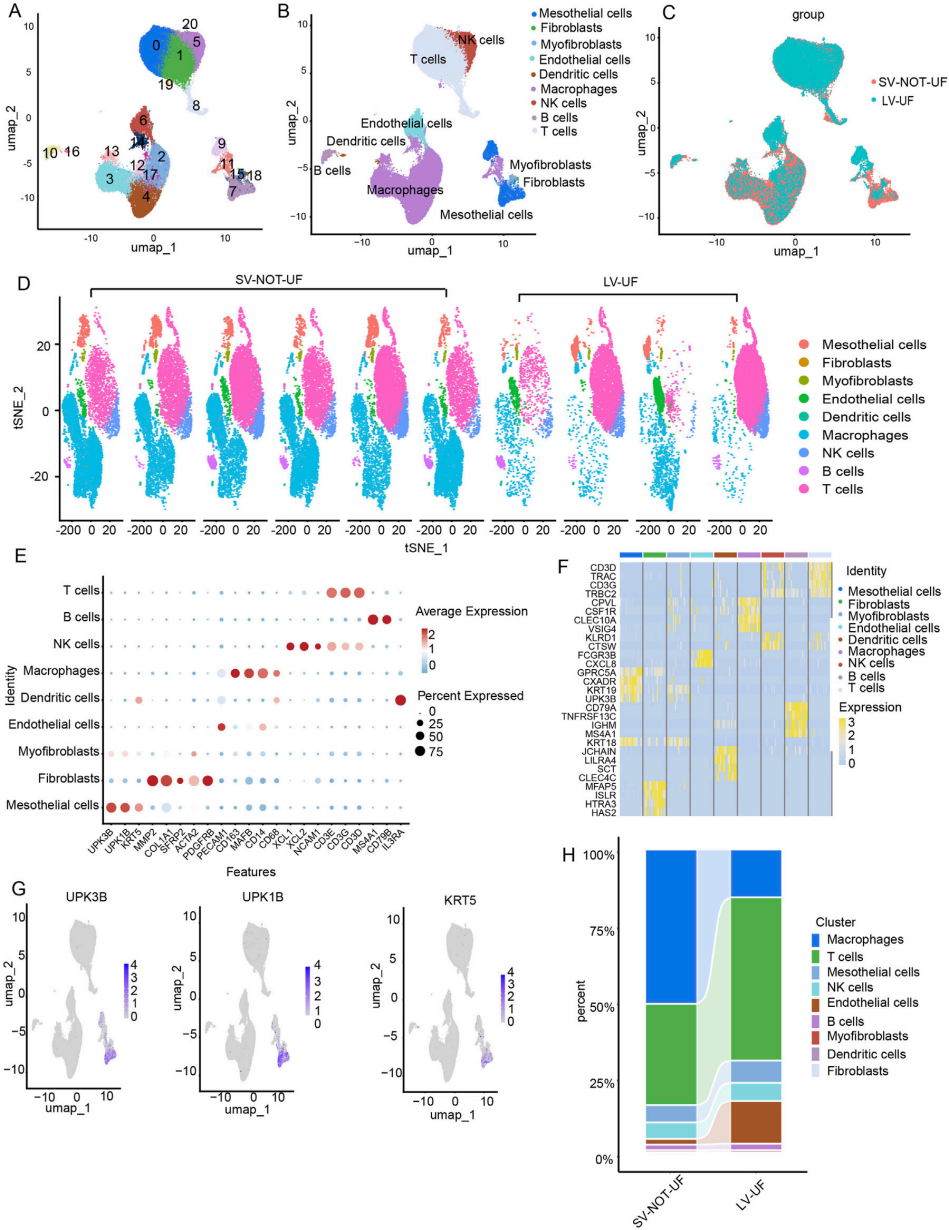
An Overview of the single-cell RNA atlas of patients undergoing PD with short vintage without ultrafiltration failure (SV-NOT-UF) and long vintage with ultrafiltration failure (LV-UF). **(A)** The Umap plot depicts the single-cell samples clustered into 21 distinct clusters. **(B)** Nine cell types identified based on the expression of marker genes. **(C)** A Umap clustering plot comparing SV-NOT-UF group and LV-UF group. **(D)** The Umap clustering plot of 10 samples. **(E)** Bubble plot displaying the expression of marker genes across 9 cell types. **(F)** A heatmap showing the differential gene expression across 9 cell types. **(G)** The Umap plot highlights the expression patterns of mesothelial cell type marker genes. **(H)** The cellular composition of SV-NOT-UF and LV-UF samples in the scRNA-seq dataset.

The flowchart of the present study was illustrated in Figure 1. We first analyzed a single-cell RNA-seq (GSE248762) in effluent obtained from six patients undergoing PD with short vintage (SV-NOT-UF) and four patients with long vintage with UF (LV- UF). Cluster analysis divided 69,188 cells into 21 clusters (Figure 2A), which through marker genes (Figure 2E) could be assigned to 9 major cell populations (Figure 2B). Those were T cells (27,252 cells), macrophages (27,961 cells), NK cells (3,829 cells), endothelial cells (3,827 cells), mesothelial cells (4,297 cells), B cells (1,297 cells), myofibroblasts (489 cells), dendritic cells (165 cells), and fibroblast cells (71 cells). The single-cell transcriptome maps of SV-NOT-UF and LV-UF samples were depicted in Figure 2C, with Figure 2D exhibiting the single-cell transcriptome maps of various samples. Heat maps illustrated variations in gene expression among 9 cell types (Figure 2F). As the first physiological and permeability screen for PD fluid to enter the body, peritoneal mesothelial cells play a vital role in the process of peritoneal fibrosis. Hence, our forthcoming investigation will center on the involvement of peritoneal mesothelial cells. In Figure 2G Umap, the expression pattern of peritoneal mesothelial marker genes was showcased. As shown in Figure 2H, the proportion of peritoneal mesothelial cells increased in the LV-UF group.

**FIGURE 3:**
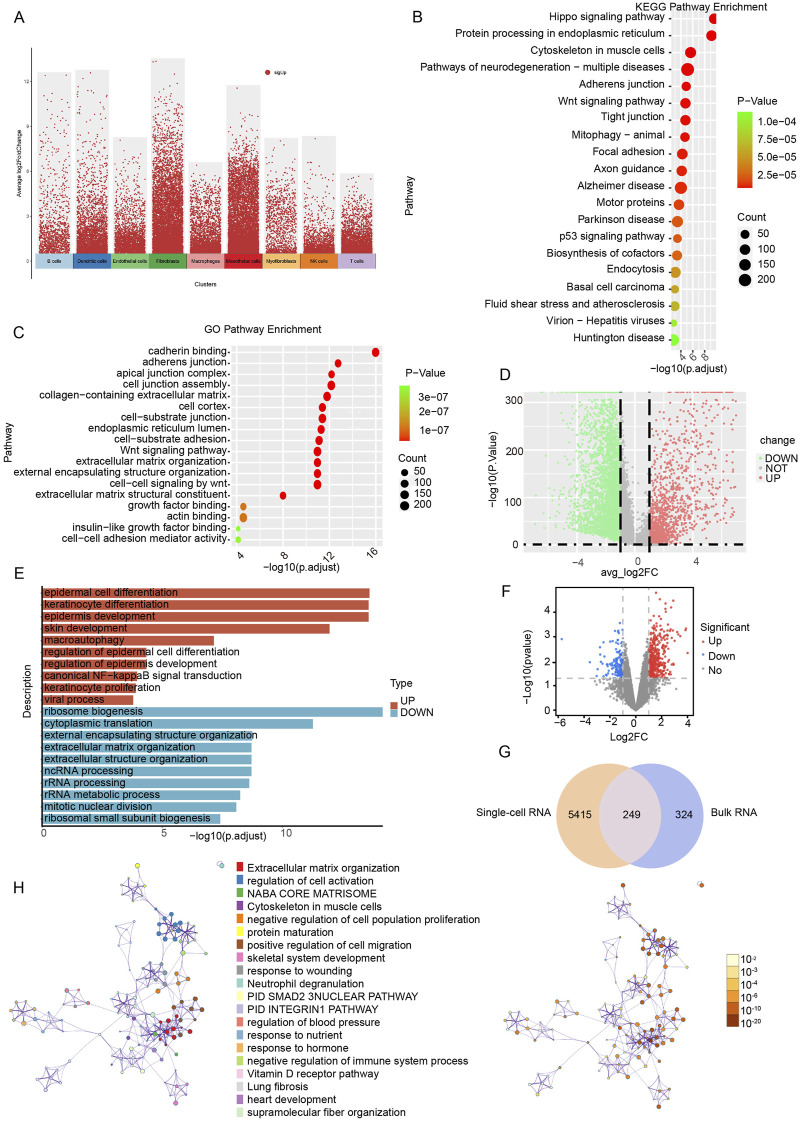
Functional enrichment analysis based on single-cell RNA and bulk RNA sequencing. **(A)** A volcano plot of differential analysis among overall cellular subgroups. Red dots represent genes that are specifically expressed in a cell cluster compared to other cell clusters. **(B)** KEGG functional enrichment map of intercellular differential genes in peritoneal mesothelial cells. **(C)** GO functional enrichment map of intercellular differential genes in peritoneal mesothelial cells. **(D)** Volcanic map of peritoneal mesothelial cell differences between groups through single-cell RNA sequencing. Red indicates upregulated genes, and green indicates downregulated genes. **(E)** Intergroup differential gene GO functional enrichment bar diagram of peritoneal mesothelial cells. **(F)** Volcanic map of peritoneal mesothelial cell differences between groups through bulk RNA sequencing. Red indicates upregulated genes, and blue indicates downregulated genes. **(G)** A total of 249 peritoneal fibrosis-related differential genes were identified by intersection of single-cell RNA transcribed differential genes and bulk RNA sequenced differential genes. **(H)** The network of KEGG enriched pathways and processes are colored by cluster ID; nodes that have the same cluster ID are typically close to each other and are colored by *P*-value.

To elucidate the biological function of mesothelial cell-associated DEGs (Figure 3A), GO and KEGG enrichment analyses were conducted. The KEGG analysis revealed the top 20 enriched pathways in the marker genes, including tight junction, mitophagy, the p53 signaling pathway and endocytosis (Figure 3B). The GO enrichment results indicated associations with adherens junction, cell junction assembly, collagen-containing extracellular matrix (ECM), ECM organization, growth factor binding, and actin binding (Figure 3C). Examining the differential genes and functions of mesothelial cells in the LV-UF and SV-NOT-UF groups, we identified 3,780 downregulated genes and 1884 upregulated genes in the volcanic map of the LV-UF group relative to the SV-NOT-UF group (Figure 3D). GO enrichment analysis revealed upregulation of epidermal cell differentiation and NF-kappaB signal transduction pathways in the mesothelial cell of the LV-UF group. Conversely, ribosome biogenesis, cytoplasmic translation, and ncRNA processing pathways were downregulated (Figure 3E).

We also conducted a comparative analysis of bulk RNA-seq data obtained from peritoneal biopsies of four patients afflicted with EPS and four uremic patients not undergoing PD (CTL group). We aimed to identify DEGs. The detailed baseline clinical characteristics could be found as previously reported (Reimold et al., 2013). DEGs were identified based on the criteria of |log (fold change)| ≥1 and *P* < 0.05 (Supplementary File 2). A volcano plot illustrated 146 downregulated genes and 427 upregulated genes in the EPS group compared to the CTL group (Figure 3F).

To identify differential genes associated with peritoneal fibrosis, we integrated the results of single-cell RNA-seq of peritoneal mesothelial cells with those of bulk RNA-seq, resulting in the identification of 249 genes as potential targets for the disease (Figure 3G). Through KEGG functional enrichment analysis, it was found that the disease targets were connected to processes such as ECM organization, cell activation, the PID SMAD2 3 NUCLEAR PATHWAY, and the vitamin D receptor (VDR) pathway (Figure 3H).

### Identification of SAL drug targets

**FIGURE 4:**
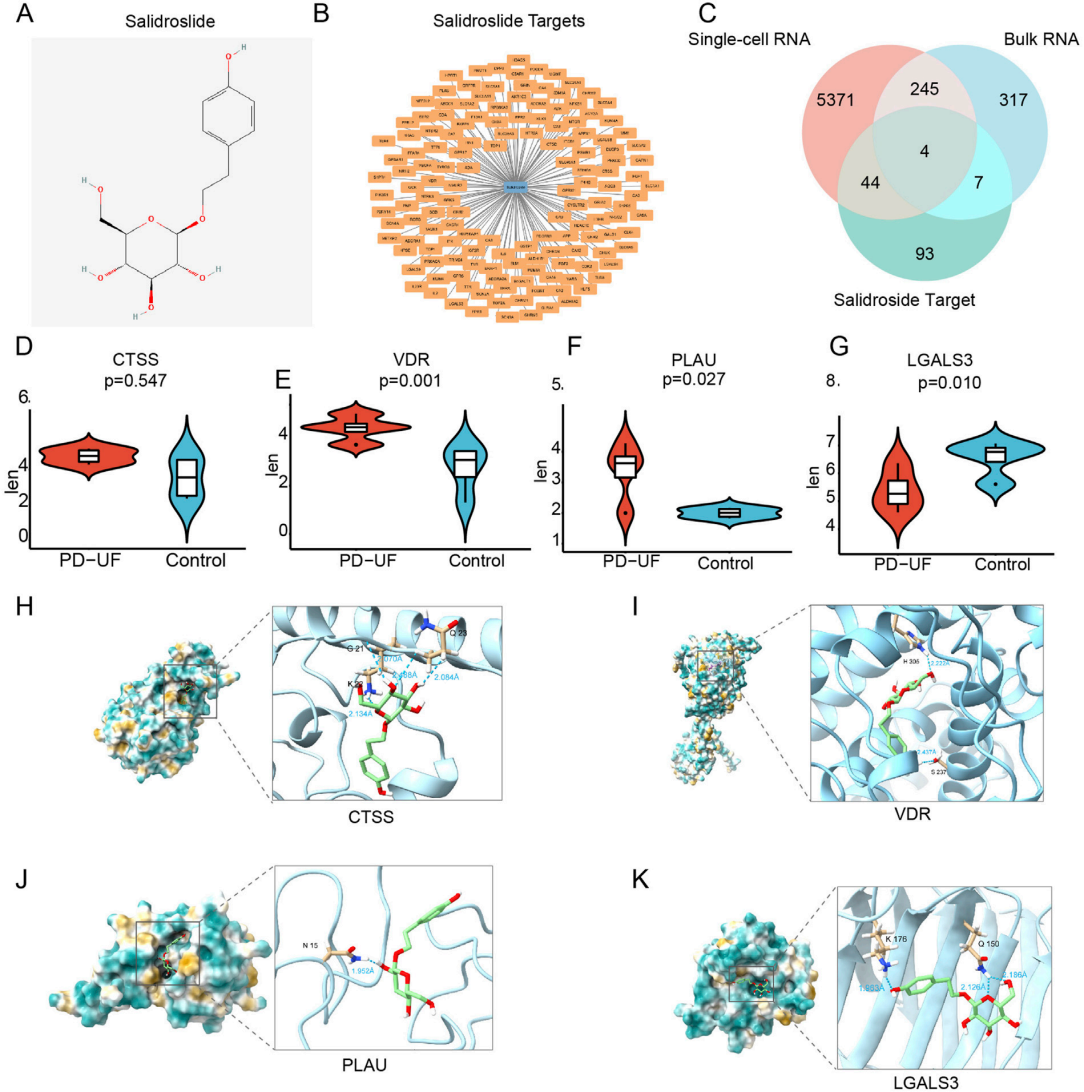
Identification of therapeutic targets for SAL against PD-related peritoneal fibrosis. **(A)** Molecular structure of SAL. **(B)** SAL target network. The node in blue is SAL, and nodes in orange are candidate targets of SAL. **(C)** Venn diagram of 4 therapeutic targets for SAL against peritoneal fibrosis. **(D–G)** The expression of 4 therapeutic targets from bulk RNA sequencing. **(H–K)** Molecular docking between SAL and 4 targets.

In the Swiss Target Prediction, SuperPred, and SEA databases, there were 17, 101 and 40 SAL drug targets, respectively (Supplementary File 1). Following the consolidation and de-duplication of these target lists, a total of 148 distinct drug targets were chosen for further investigation (Figures 4A,B).

### Identification of therapeutic targets for SAL against peritoneal fibrosis

By intersecting the drug targets associated with SAL with the targets related to peritoneal fibrosis, we identified potential therapeutic targets for SAL against peritoneal fibrosis, identifying a total of 4 targets (Figure 4C): cathepsin S (CTSS), VDR, plasminogen activator urokinase (PLAU), and galectin 3 (LGALS3). Furthermore, PLAU and VDR displayed reduced expression levels in the CTL group but elevated expression levels in the EPS group (Figures 4E,F). Conversely, LGALS3 showed increased expression in the CTL group and decreased expression in the EPS group (Figure 4G). There was no notable effect on the expression of CTSS (Figure 4D). We proceeded to analyze the binding affinity and mechanism of SAL with 4 specific targets using molecular docking with the AutoDock Vina software (Figures 4H–K).

### SAL suppressed the production of the ECM *in vitro*

**FIGURE 5:**
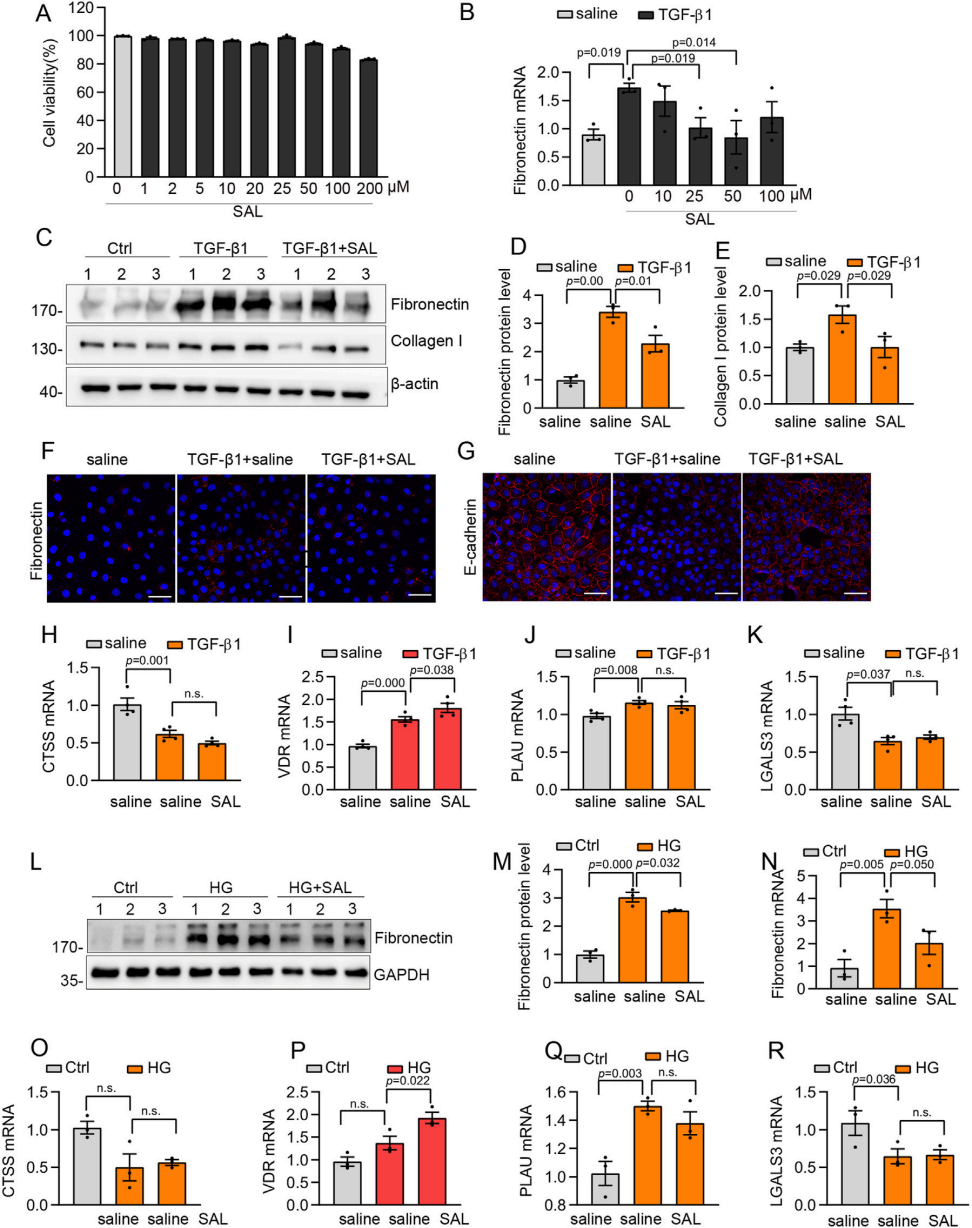
SAL inhibited the production of the extracellular matrix and increased VDR expression *in vitro*. **(A)** The viability of Met5-A cells incubated with 0–200 μM SAL. **(B)** The mRNA expression levels of Fibronectin in different groups as indicated. **(C)** Western blot analysis showed expression of Collagen I and Fibronectin in different groups as indicated. **(D,E)** Quantitative analysis of Collagen I and Fibronectin. **(F,G)** Representative images for immunofluorescence staining of E-cadherin and Fibronectin in Met5-A cells after treatment with TGF-β1 (5 ng/mL) and saline or SAL for 48 h **(H–K)** The mRNA expression levels of LGALS3, CTSS, PLAU and VDR in different groups as indicated. **(L)** Western blot analysis showed expression of Fibronectin in different groups as indicated. **(M)** Quantitative analysis of Fibronectin. **(N–R)** The mRNA expression levels of Fibronectin, LGALS3, CTSS, PLAU and VDR in different groups as indicated.

Based on the prediction of network pharmacology analysis and molecular docking, we assessed the effects of SAL on TGF-β1-induced ECM production in human mesothelial cells (Met5-A) in an *in vitro* environment. Concentrations of SAL below 200 μM did not have a significant impact on cell viability (Figure 5A). Besides, the expression of Fibronectin was decreased with SAL at concentrations of 25 and 50 μM in a TGF-β1 environment (Figure 5B). Consequently, we selected 50 μM SAL for the ensuing experiments.

Treatment with SAL significantly alleviated the increased expression of Fibronectin and Collagen I induced by TGF-β1 (Figures 5C–E). Besides, immunofluorescence staining showed that SAL treatment led to a reduction in the deposition of Fibronectin and rescued the expression of E-cadherin induced by TGF-β1 (Figures 5F,G). In addition to TGF-β1, which has been identified as the primary molecule in the development of peritoneal dysfunction and damage to peritoneal mesothelial cells (Huang et al., 2023; Si et al., 2019; Mo et al., 2023), high glucose also plays a crucial role in causing damage to mesothelial cells and peritoneal fibrosis (Krediet and Parikova, 2022; Hu et al., 2023). Therefore, we investigated the impact of SAL in a high glucose environment (138 mmol/L) on peritoneal mesothelial cells. SAL treatment effectively mitigated the upregulation of Fibronectin induced by high glucose levels (Figures 5L–N).

### Effect of SAL on target proteins

To assess the potential impact of the predicted targets on the improvement of peritoneal fibrosis, we conducted RT-qPCR analyses on the four specific targets. After exposure to TGF-β1, there was a significant increase in the mRNA expression levels of PLAU and VDR, whereas CTSS and LGALS3 exhibited a marked downregulation (Figures 5H–K). SAL treatment increased VDR expression after exposure to TGF-β1 (Figure 5I). Furthermore, under high-glucose conditions, SAL treatment led to an increase in VDR expression without significantly affecting the expression of CTSS, LGALS3, and PLAU (Figures 5O–R).

### SAL attenuated PD-related peritoneal fibrosis *in vivo*

**FIGURE 6:**
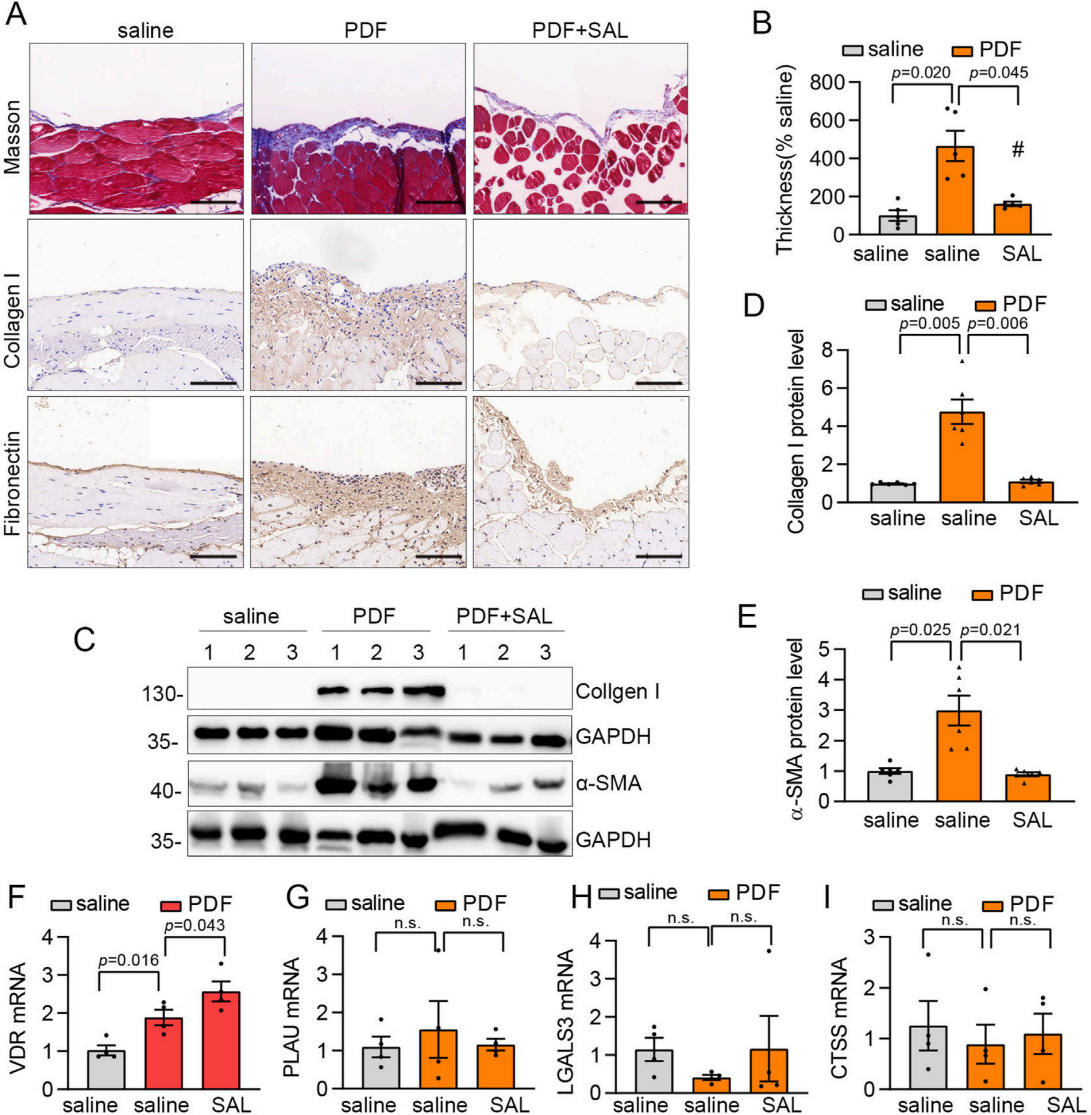
SAL attenuated PD-related peritoneal fibrosis in mice. **(A)** Representative micrographs show collagen deposition by Masson’s trichrome staining (Scale bar, 50 μm), Fibronectin and Collagen I detected by immunohistochemical staining (Scale bar, 50 μm) in the parietal peritoneum in different groups as indicated. **(B)** Quantitative analysis of the peritoneal thickness by Masson’s trichrome staining. n = 5. **(C)** Western blot analysis shows visceral peritoneum expression of Collagen I and a-SMA in different groups as indicated. **(D,E)** Quantitative analysis of Collagen I and α-SMA. n = 6. **(F–I)** The mRNA expression levels of LGALS3, CTSS, PLAU and VDR in different groups as indicated. n = 4.

In the upcoming investigation, we explored the efficacy of SAL treatment in reducing PD-related peritoneal fibrosis in mice. A mouse model of peritoneal fibrosis was induced by daily intraperitoneal administration of 3 ml of standard PD fluid containing 4.25% glucose. PD mice were subjected to daily intragastric administration of 80 mg/kg SAL, while the control group received saline. Masson’s trichrome staining revealed thickened matrix deposition in PD mice compared to the normal group, a condition that was mitigated by SAL treatment (Figures 6A,B). Enhanced synthesis of ECM-related proteins, including Collagen I and Fibronectin, were evident in PD mice with SAL effectively suppressing their expression levels (Figure 6A). Furthermore, immunoblot analysis demonstrated marked attenuation of PD-induced expression of Collagen I and α-SMA by SAL (Figures 6C–E). Furthermore, RT-qPCR analyses were performed on the four specific targets *in vivo*. The upregulation of VDR was observed in PD mice, with SAL treatment effectively augmenting its expression level (Figure 6F). However, there was no notable effect on the expression of CTSS, LGALS3, and PLAU (Figures 6G–I).

## Discussion

PD-related peritoneal fibrosis is characterized by chronic, non-suppurative inflammation and excessive fibrous tissue growth in the peritoneal membrane. This process disrupts the tissue’s integrity and function, ultimately leading to peritoneal dysfunction. Although the addition of Ala-Gln to PD fluids (Ferrantelli et al., 2016), as well as the use of newer PD solutions like icodextrin (Nakao et al., 2010), may attenuate the development of peritoneal fibrosis, their safety, feasibility, and cost effectiveness need to be further investigated. Hence, the search for a pharmaceutical agent exhibiting favorable efficacy and minimal adverse effects is imperative to retard the progression of peritoneal fibrosis.

Our analysis of a single-cell dataset with 69,188 cells from four LV-UF and six SV-NOT-UF PD patient pairs revealed that macrophages and T cells were the predominant cell types. Their elaborate interactions with other cells are essential in immune regulation and fibrosis (Terri et al., 2021; Diao et al., 2024; Helmke et al., 2019). Persistent low-grade inflammation is a significant factor in the development of structural alterations in the peritoneum, resulting in fibrin deposition, vascular proliferation, and the formation of a fibrous capsule. As PD progresses from SV-NOT-UF to LV-UF, there is a gradual decrease in macrophages, possibly anti-inflammatory M2-like macrophages, because studies have indicated that M2-like macrophage reperfusion reduces histological damage, ECM deposition, and peritoneal ultrafiltration functional decline in mice compared to the M1-like macrophage (Li et al., 2018; Chu et al., 2025). This suggests that the local macrophage system in the peritoneum undergoes dynamic changes. T cells-mesothelial crosstalk also plays a significant role in peritoneal fibrosis, with studies showing that blocking this interaction can mitigate peritoneal fibrosis (Sun et al., 2025).

This study focuses on peritoneal mesothelial cells because they act as the primary physiological barrier for the entry of peritoneal dialysate into the patient’s body, affecting the development and progression of peritoneal fibrosis (Si et al., 2019; Diao et al., 2024). Within this dataset, we successfully distinguished a total of 4,297 mesothelial cells following cell type classification. Our findings indicate the involvement of mesothelial cells in the activation of crucial pathways, including the Wnt signaling, autophagy, and Hippo signaling pathways, which is supported by previous research (Li et al., 2024; Zheng et al., 2024; Zhu et al., 2021). Moreover, in the context of peritoneal fibrosis, peritoneal mesothelial cells have been observed to activate the NF-kappaB pathway, consistent with the discoveries of Zhang et al. (2022). In our study, we innovatively employed a multi-omics strategy to pinpoint crucial targets of peritoneal mesothelial cells implicated in peritoneal fibrosis, uncovering a total of 289 potential key targets. Functional enrichment analysis of these targets revealed their association with ECM and other pathways. Notably, our investigation highlighted the involvement of the VDR pathway, a finding supported by subsequent experimental validation. The documented role of the VDR pathway in peritoneal fibrosis further underscores its significance (Yang et al., 2017; Liu et al., 2019).

SAL is a promising drug with low toxicity and few side effects, with a wide range of pharmacological properties, including encompassing anti-inflammatory, anti-fibrotic, anti-cancer, and antioxidant properties. Nevertheless, it remains uncertain whether SAL exerts a therapeutic influence on PD-related peritoneal fibrosis. In this study, we observed a significant reduction in ECM deposition and enhanced peritoneal thickening in mice treated with SAL compared to those treated with the vehicle. Additionally, SAL reduced the accumulation of ECM in peritoneal mesothelial cells induced by TGFβ1 and high glucose. Our findings suggest that SAL holds significant clinical promise and could serve as a viable therapeutic approach for peritoneal fibrosis.

In order to explore the protective effects of SAL on peritoneal fibrosis, a network pharmacological analysis was conducted to pinpoint 4 compelling targets. Studies have shown that PLAU and CTSS are involved in signaling pathways related to apoptosis and cell migration, which are also implicated in peritoneal fibrosis (He et al., 2018; Luo et al., 2023). However, our findings showed that SAL does not have a significant impact on the expression of PLAU or CTSS, indicating that they are not the focus of SAL in reducing peritoneal fibrosis.

In the current study, molecular docking analyses revealed significant binding potential between SAL and VDR. We then validated the mRNA expression levels by RT-qPCR. Among 4 DEGs. VDR was increased in a mouse model of peritoneal fibrosis and *in vitro* peritoneal mesothelial cells treated with TGF-β1 and high glucose.

The VDR functions as a nuclear regulatory transcription factor. The established belief suggests that when vitamin D binds to VDR, it has the capacity to either suppress or boost the transcription of numerous genes by altering the activity of the target gene promoter (Li X. et al., 2023). VDR-directed therapy offers a dual advantage by decreasing fibrosis and inflammation in both acute and chronic murine pancreatitis (Sherman et al., 2014). Numerous studies indicate the involvement of vitamin D/VDR in diverse renal diseases. Activation of VDR may aid in the restoration of mitochondrial function in renal tubular epithelial cells under diabetic nephropathy conditions (Chen et al., 2024). VDR partially restored defective autophagy and reduced inflammation in the kidneys of STZ-induced diabetic mice (Li et al., 2022), while autophagy also plays an emerging role in peritoneal fibrosis (Li S. et al., 2019). Besides, targeting VDR inhibits epithelial to mesenchymal transition in human peritoneal mesothelial cells. These findings suggest that VDR could be a potential protective target in peritoneal fibrosis. Furthermore, it was interestingly observed that SAL could enhance VDR expression, highlighting the importance of VDR as a key target in alleviating peritoneal fibrosis.

The current study’s strengths lie in the comprehensive identification of therapeutic targets for SAL against PD-related peritoneal fibrosis through a multi-omics strategy and network pharmacology analysis. The identified targets were validated through molecular docking simulations and *in vitro* and *in vivo* experiments. To our knowledge, no published studies have conducted a comprehensive screening and validation of therapeutic targets for SAL against PD-related peritoneal fibrosis. These findings comprehensively identify potential therapeutic targets for SAL in combating peritoneal fibrosis, offering a theoretical foundation for utilizing SAL in treating PD-related peritoneal fibrosis. Undeniably, some limitations also existed in our study. First, our investigation solely focused on examining the anti-fibrosis properties of SAL, without delving into its potential role in modulating inflammation. Secondly, Experimentation on the pathway implicated in SAL targeting VDR is needed. Further tests will be carried out in the future to delve into the molecular pathways responsible for the anti-fibrotic impact of SAL in peritoneal fibrosis in more depth.

## Conclusion

By employing multi-omics analysis, network pharmacology, and experimental validation, we have established that SAL exerts a notable anti-fibrotic influence on PD-related peritoneal fibrosis. The underlying protective mechanism may entail the modulation of VDR domains to mitigate ECM accumulation. These results collectively pinpoint potential therapeutic avenues for SAL in combatting peritoneal fibrosis, providing a theoretical basis for its clinical efficacy in this specific condition.

## Data Availability

The datasets presented in this study can be found in online repositories. The names of the repository/repositories and accession number(s) can be found in the article/Supplementary Material.

## References

[B1] Chen Y. Li K. Zhao H. Hao Z. Yang Y. Gao M. (2022). Integrated lipidomics and network pharmacology analysis to reveal the mechanisms of berberine in the treatment of hyperlipidemia. J. Transl. Med. 20 (1), 412. 10.1186/s12967-022-03623-0 36076294 PMC9461205

[B2] Chen H. Zhu J. Le Y. Pan J. Liu Y. Liu Z. (2022). Salidroside inhibits doxorubicin-induced cardiomyopathy by modulating a ferroptosis-dependent pathway. Phytomedicine 99, 153964. 10.1016/j.phymed.2022.153964 35180677

[B3] Chen H. Zhang H. Li A. M. Liu Y. T. Liu Y. Zhang W. (2024). Vdr regulates mitochondrial function as a protective mechanism against renal tubular cell injury in diabetic rats. Redox Biol. 70, 103062. 10.1016/j.redox.2024.103062 38320454 PMC10850784

[B4] Chu C. Huang Y. Cao L. Ji S. Zhu B. Shen Q. (2025). Role of macrophages in peritoneal dialysis-associated peritoneal fibrosis. Ren. Fail. 47 (1), 2474203. 10.1080/0886022X.2025.2474203 40044628 PMC11884102

[B5] Diao X. Zhan C. Ye H. Wu H. Yi C. Lin J. (2024). Single-cell transcriptomic reveals the peritoneal microenvironmental change in long-term peritoneal dialysis patients with ultrafiltration failure. iScience 27 (12), 111383. 10.1016/j.isci.2024.111383 39687014 PMC11647153

[B6] Ferrantelli E. Liappas G. Vila C. M. Keuning E. D. Foster T. L. Vervloet M. G. (2016). The dipeptide alanyl-glutamine ameliorates peritoneal fibrosis and attenuates il-17 dependent pathways during peritoneal dialysis. Kidney Int. 89 (3), 625–635. 10.1016/j.kint.2015.12.005 26880457

[B7] Hai Z. Wu Y. Ning Z. (2023). Salidroside attenuates atrial fibrosis and atrial fibrillation vulnerability induced by angiotensin-ii through inhibition of loxl2-tgf-β1-smad2/3 pathway. Heliyon 9 (11), e21220. 10.1016/j.heliyon.2023.e21220 37920527 PMC10618763

[B8] He Y. Tsou P. S. Khanna D. Sawalha A. H. (2018). Methyl-cpg-binding protein 2 mediates antifibrotic effects in scleroderma fibroblasts. Ann. Rheum. Dis. 77 (8), 1208–1218. 10.1136/annrheumdis-2018-213022 29760157 PMC7297461

[B9] Helmke A. Nordlohne J. Balzer M. S. Dong L. Rong S. Hiss M. (2019). Cx3cl1-cx3cr1 interaction mediates macrophage-mesothelial cross talk and promotes peritoneal fibrosis. Kidney Int. 95 (6), 1405–1417. 10.1016/j.kint.2018.12.030 30948201

[B10] Himmelfarb J. Vanholder R. Mehrotra R. Tonelli M. (2020). The current and future landscape of dialysis. Nat. Rev. Nephrol. 16 (10), 573–585. 10.1038/s41581-020-0315-4 32733095 PMC7391926

[B11] Hu M. Zhang D. Xu H. Zhang Y. Shi H. Huang X. (2021). Salidroside activates the amp-activated protein kinase pathway to suppress nonalcoholic steatohepatitis in mice. Hepatology 74 (6), 3056–3073. 10.1002/hep.32066 34292604

[B12] Hu W. Li G. Dong W. He P. Liu W. Wu Y. (2023). Single-cell sequencing reveals peritoneal environment and insights into fibrosis in capd patients. iScience 26 (4), 106336. 10.1016/j.isci.2023.106336 36968085 PMC10034447

[B13] Huang Q. Sun Y. Peng L. Sun J. Sha Z. Lin H. (2023). Extracellular vesicle-packaged ilk from mesothelial cells promotes fibroblast activation in peritoneal fibrosis. J. Extracell. Vesicles 12 (7), e12334. 10.1002/jev2.12334 37357686 PMC10291285

[B14] Krediet R. T. Parikova A. (2022). Relative contributions of pseudohypoxia and inflammation to peritoneal alterations with long-term peritoneal dialysis patients. Clin. J. Am. Soc. Nephrol. 17 (8), 1259–1266. 10.2215/CJN.15371121 35168992 PMC9435980

[B15] Li Q. Zheng M. Liu Y. Sun W. Shi J. Ni J. (2018). A pathogenetic role for m1 macrophages in peritoneal dialysis-associated fibrosis. Mol. Immunol. 94, 131–139. 10.1016/j.molimm.2017.12.023 29306153

[B16] Li R. Guo Y. Zhang Y. Zhang X. Zhu L. Yan T. (2019). Salidroside ameliorates renal interstitial fibrosis by inhibiting the tlr4/nf-κb and mapk signaling pathways. Int. J. Mol. Sci. 20 (5), 1103. 10.3390/ijms20051103 30836660 PMC6429495

[B17] Li S. Peng F. Gong W. Wu J. Wang Y. Xu Z. (2019). Dimethylaminomicheliolide ameliorates peritoneal fibrosis through the activation of autophagy. J. Mol. Med. 97 (5), 659–674. 10.1007/s00109-019-01757-1 30854581

[B18] Li A. Yi B. Han H. Yang S. Hu Z. Zheng L. (2022). Vitamin d-vdr (vitamin d receptor) regulates defective autophagy in renal tubular epithelial cell in streptozotocin-induced diabetic mice via the ampk pathway. Autophagy 18 (4), 877–890. 10.1080/15548627.2021.1962681 34432556 PMC9037529

[B19] Li S. S. Luo C. Chen S. Zhuang Y. Ji Y. Zeng Y. (2023). Brahma-related gene 1 acts as a profibrotic mediator and targeting it by micheliolide ameliorates peritoneal fibrosis. J. Transl. Med. 21 (1), 639. 10.1186/s12967-023-04469-w 37726857 PMC10510267

[B20] Li X. X. Fan Q. L. Ma T. K. Liu C. Shi H. Sun Y. Y. (2023). Myct1 attenuates renal fibrosis and tubular injury in diabetic kidney disease. iScience 26 (9), 107609. 10.1016/j.isci.2023.107609 37664593 PMC10470386

[B21] Li S. Zhuang Y. Ji Y. Chen X. He L. Chen S. (2024). Brg1 accelerates mesothelial cell senescence and peritoneal fibrosis by inhibiting mitophagy through repression of oxr1. Free Radic. Biol. Med. 214, 54–68. 10.1016/j.freeradbiomed.2024.01.054 38311259

[B22] Liang Q. Guo R. Tsao J. R. He Y. Wang C. Jiang J. (2023). Salidroside alleviates oxidative stress in dry eye disease by activating autophagy through ampk-sirt1 pathway. Int. Immunopharmacol. 121, 110397. 10.1016/j.intimp.2023.110397 37302369

[B23] Liu K. H. Fu J. Zhou N. Yin W. Yang Y. Y. Ouyang S. X. (2019). 1,25-dihydroxyvitamin d3 prevents epithelial-mesenchymal transition of hmrsv5 human peritoneal mesothelial cells by inhibiting histone deacetylase 3 (hdac3) and increasing vitamin d receptor (vdr) expression through the wnt/β-catenin signaling pathway. Med. Sci. Monit. 25, 5892–5902. 10.12659/MSM.916313 31391414 PMC6698096

[B24] Liu Q. Chen J. Zeng A. Song L. (2023). Pharmacological functions of salidroside in renal diseases: facts and perspectives. Front. Pharmacol. 14, 1309598. 10.3389/fphar.2023.1309598 38259279 PMC10800390

[B25] Luo S. Zhao X. Jiang J. Deng B. Liu S. Xu H. (2023). Piezo1 specific deletion in macrophage protects the progression of liver fibrosis in mice. Theranostics 13 (15), 5418–5434. 10.7150/thno.86103 37908726 PMC10614683

[B26] Mo M. Zeng Y. Zeng Y. Li S. He X. Chen X. (2023). N-methylpiperazine-diepoxyovatodiolide ameliorates peritoneal fibrosis via suppressing tgf-β/smad and jak/stat signaling pathway. Chem.-Biol. Interact. 382, 110589. 10.1016/j.cbi.2023.110589 37268199

[B27] Nakao A. Nakao K. Takatori Y. Kojo S. Inoue J. Akagi S. (2010). Effects of icodextrin peritoneal dialysis solution on the peritoneal membrane in the stz-induced diabetic rat model with partial nephrectomy. Nephrol. Dial. Transpl. 25 (5), 1479–1488. 10.1093/ndt/gfp479 19759273

[B28] Nogales C. Mamdouh Z. M. List M. Kiel C. Casas A. I. Schmidt H. (2022). Network pharmacology: curing causal mechanisms instead of treating symptoms. Trends Pharmacol. Sci. 43 (2), 136–150. 10.1016/j.tips.2021.11.004 34895945

[B29] Panossian A. Wikman G. Sarris J. (2010). Rosenroot (rhodiola rosea): traditional use, chemical composition, pharmacology and clinical efficacy. Phytomedicine 17 (7), 481–493. 10.1016/j.phymed.2010.02.002 20378318

[B30] Pletinck A. Vanholder R. Veys N. Van Biesen W. (2012). Protecting the peritoneal membrane: factors beyond peritoneal dialysis solutions. Nat. Rev. Nephrol. 8 (9), 542–550. 10.1038/nrneph.2012.144 22777203

[B31] Reimold F. R. Braun N. Zsengellér Z. K. Stillman I. E. Karumanchi S. A. Toka H. R. (2013). Transcriptional patterns in peritoneal tissue of encapsulating peritoneal sclerosis, a complication of chronic peritoneal dialysis. PLoS One 8 (2), e56389. 10.1371/journal.pone.0056389 23418565 PMC3572070

[B32] Shang L. Wang Y. Li J. Zhou F. Xiao K. Liu Y. (2023). Mechanism of sijunzi decoction in the treatment of colorectal cancer based on network pharmacology and experimental validation. J. Ethnopharmacol. 302 (Pt A), 115876. 10.1016/j.jep.2022.115876 36343798

[B33] Sherman M. H. Yu R. T. Engle D. D. Ding N. Atkins A. R. Tiriac H. (2014). Vitamin d receptor-mediated stromal reprogramming suppresses pancreatitis and enhances pancreatic cancer therapy. Cell 159 (1), 80–93. 10.1016/j.cell.2014.08.007 25259922 PMC4177038

[B34] Si M. Wang Q. Li Y. Lin H. Luo D. Zhao W. (2019). Inhibition of hyperglycolysis in mesothelial cells prevents peritoneal fibrosis. Sci. Transl. Med. 11 (495), eaav5341. 10.1126/scitranslmed.aav5341 31167927

[B35] Sun Y. Huang Q. Sun J. Zhou H. Guo D. Peng L. (2025). Mucosal-associated invariant t (mait) cell-mediated immune mechanisms of peritoneal dialysis-induced peritoneal fibrosis and therapeutic targeting. J. Am. Soc. Nephrol. 10.1681/ASN.0000000627 PMC12147976 39874111

[B36] Tang H. Gao L. Mao J. He H. Liu J. Cai X. (2016). Salidroside protects against bleomycin-induced pulmonary fibrosis: activation of nrf2-antioxidant signaling, and inhibition of nf-κb and tgf-β1/smad-2/-3 pathways. Cell Stress Chaperones 21 (2), 239–249. 10.1007/s12192-015-0654-4 26577463 PMC4786523

[B37] Tao H. Wu X. Cao J. Peng Y. Wang A. Pei J. (2019). Rhodiola species: a comprehensive review of traditional use, phytochemistry, pharmacology, toxicity, and clinical study. Med. Res. Rev. 39 (5), 1779–1850. 10.1002/med.21564 30652331

[B38] Terri M. Trionfetti F. Montaldo C. Cordani M. Tripodi M. Lopez-Cabrera M. (2021). Mechanisms of peritoneal fibrosis: focus on immune cells-peritoneal stroma interactions. Front. Immunol. 12, 607204. 10.3389/fimmu.2021.607204 33854496 PMC8039516

[B39] Xue H. Li P. Luo Y. Wu C. Liu Y. Qin X. (2019). Salidroside stimulates the sirt1/pgc-1α axis and ameliorates diabetic nephropathy in mice. Phytomedicine 54, 240–247. 10.1016/j.phymed.2018.10.031 30668374

[B40] Yang L. Fan Y. Zhang X. Ma J. (2017). Mirna-23 regulates high glucose induced epithelial to mesenchymal transition in human mesotheial peritoneal cells by targeting vdr. Exp. Cell Res. 360 (2), 375–383. 10.1016/j.yexcr.2017.09.029 28942023

[B41] Zhang J. Chen Y. Chen T. Miao B. Tang Z. Hu X. (2021). Single-cell transcriptomics provides new insights into the role of fibroblasts during peritoneal fibrosis. Clin. Transl. Med. 11 (3), e321. 10.1002/ctm2.321 33784014 PMC7908046

[B42] Zhang X. Xie L. Long J. Xie Q. Zheng Y. Liu K. (2021). Salidroside: a review of its recent advances in synthetic pathways and pharmacological properties. Chem.-Biol. Interact. 339, 109268. 10.1016/j.cbi.2020.109268 33617801

[B43] Zhang Y. Feng W. Peng X. Zhu L. Wang Z. Shen H. (2022). Parthenolide alleviates peritoneal fibrosis by inhibiting inflammation via the nf-κb/tgf-β/smad signaling axis. Lab. Invest. 102 (12), 1346–1354. 10.1038/s41374-022-00834-3 36307537

[B44] Zheng L. Chen W. Yao K. Xie Y. Liao C. Zhou T. (2024). Clinical and preclinical studies of mesenchymal stem cells to alleviate peritoneal fibrosis. Stem Cell Res. Ther. 15 (1), 237. 10.1186/s13287-024-03849-3 39080683 PMC11290310

[B45] Zhou W. Zhang H. Wang X. Kang J. Guo W. Zhou L. (2022). Network pharmacology to unveil the mechanism of moluodan in the treatment of chronic atrophic gastritis. Phytomedicine 95, 153837. 10.1016/j.phymed.2021.153837 34883416

[B46] Zhu X. Shan Y. Yu M. Shi J. Tang L. Cao H. (2021). Tetramethylpyrazine ameliorates peritoneal angiogenesis by regulating vegf/hippo/yap signaling. Front. Pharmacol. 12, 649581. 10.3389/fphar.2021.649581 33927624 PMC8076865

