# Supplementary material for: Integrating single-cell RNA-seq, bulk RNA-seq and network pharmacology reveals protective effect of salidroside in peritoneal dialysis-associated peritoneal fibrosis
Source: Front Pharmacol. 2025 Jun 20;16:1558366. doi: 10.3389/fphar.2025.1558366 (PMC12226536; doi:10.3389/fphar.2025.1558366)

**Supplementary material**

**Supplementary Table S1. The detail information of antibodies used in this study**

| Name | Manufacturer | Product code | Host | Application |
| --- | --- | --- | --- | --- |
| anti-Collagen I | BOSTE | BA0325 | Rabbit | WB,IHC |
| anti-E-cadherin | BD Biosciences | 610181 | Mouse | IF |
| anti-β-actin | EarthOx | E021020-01 | Mouse | WB |
| anti-Fibronetin | Abcam | ab2413 | Rabbit | WB,IHC,IF |
| anti-α-SMA | Abcam | ab124964 | Rabbit | WB |
| anti-GAPDH | EarthOx | E021010-03 | Mouse | WB |
| Dylight 594 anti-Mouse IgG | Abbkine | A23410 | Goat | IF |
| Dylight 594 anti- Rabbit IgG | Abbkine | A23420 | Goat | IF |

WB: western blot; IHC: immunohistochemistry; IF: immunofluorescence

**Supplementary Table S2. RT-qPCR primer sequences**

| Gene | Forward(5’-3’) | Reverse(5’-3’) |
| --- | --- | --- |
| Homo-β-actin | CCTGGCACCCAGCACAAT | GGGCCGGACTCGTCATAC |
| Homo-Fibronectin |  |  |
| Homo-CTSS | GGCATGAACCACCTGGGAGAC | GTTCTGGGCACTGAGAGACACC |
| Homo-LGALS3 | CCTACCCTGGAGCACCTGGAG | CGGTGGCACTTGGCTGTCC |
| Homo-VDR | GACGCCCACCATAAGACCTACG | GGCTCCCTCCACCATCATTCAC |
| Homo-PLAU | GTCGCTCAAGGCTTAACTCCAAC | AACGGATCTTCAGCAAGGCAATG |
| Mus-VDR | CACCACAAGACCTACGACCC | GTCGGTCTGGGGAGACAATG |
| Mus-CTSS | GAGCACCACACTTCAGGATGA | TCCCAATGGTAGTCCAGGGT |
| Mus-LGALS3 | TAATCAGGTGAGCGGCACAG | TAGGTGAGCATCGTTGACCG |
| Mus-PLAU | CATCCATCCAGTCCTTGCGT | GTGTTGGCCTTTCCTCGGTA |
| Mus-β-actin | GCAGGAGTACGATGAGTCCG | ACGCAGCTCAGTAACAGTCC |


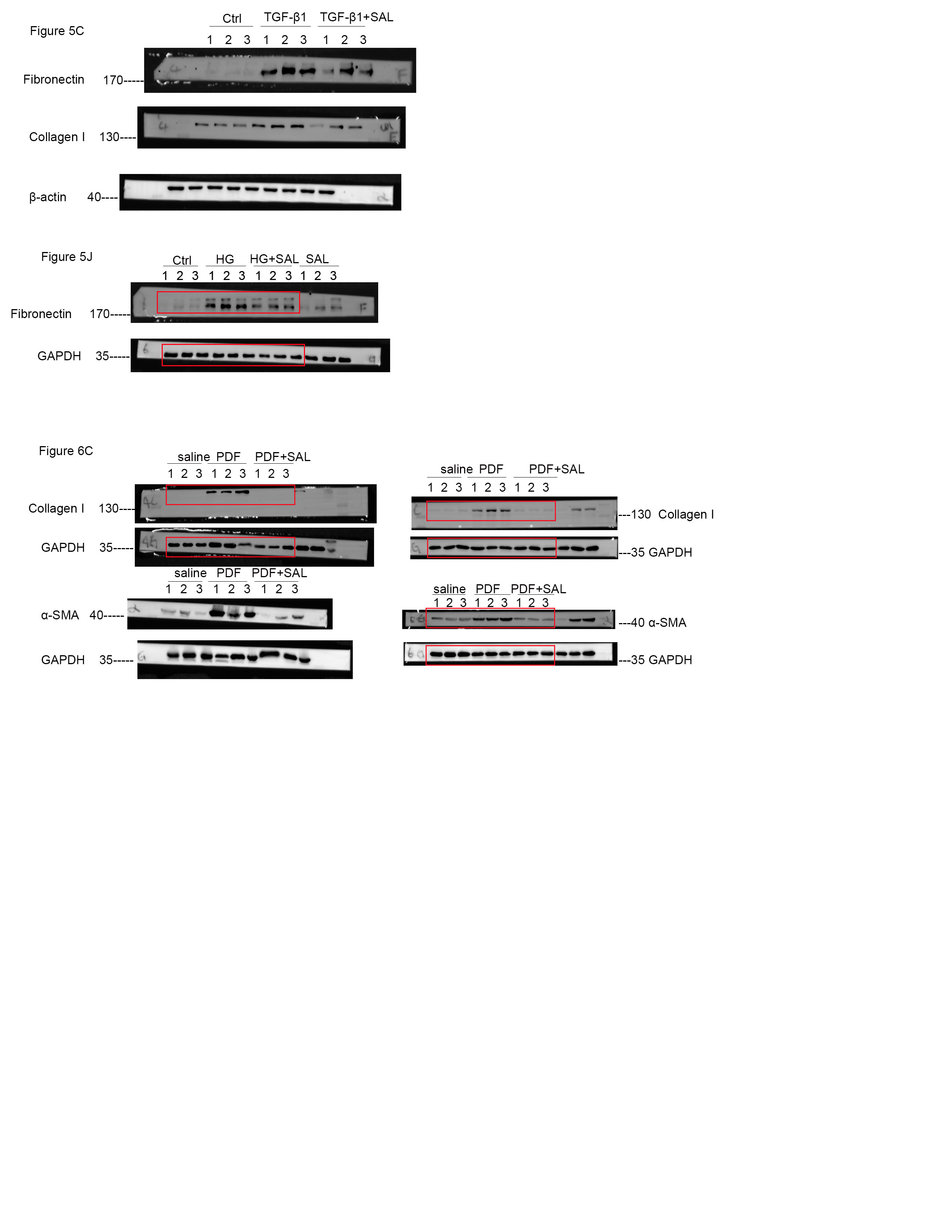

Supplement: Supplementary file 1 [file Table1.docx]
